# Cardioprotective Role of Estrogen in Takotsubo Cardiomyopathy

**DOI:** 10.7759/cureus.22845

**Published:** 2022-03-04

**Authors:** Ahsan Waqar, Ashish Jain, Christine Joseph, Kosha Srivastava, Olive Ochuba, Tasnim Alkayyali, Sujan Poudel

**Affiliations:** 1 Family Medicine, California Institute of Behavioral Neurosciences and Psychology, Fairfield, USA; 2 Internal Medicine, California Institute of Behavioral Neurosciences and Psychology, Fairfield, USA; 3 Urology and Obstetrics & Gynecology, California Institute of Behavioral Neurosciences and Psychology, Fairfield, USA; 4 Neurology, California Institute of Behavioral Neurosciences and Psychology, Fairfield, USA; 5 Pathology, California Institute of Behavioral Neurosciences and Psychology, Fairfield, USA; 6 Psychiatry and Behavioral Sciences, California Institute of Behavioral Neurosciences and Psychology, Fairfield, USA; 7 Division of Research & Academic Affairs, Larkin Community Hospital, South Miami, USA

**Keywords:** sex hormones, estradiol, estrogen, left ventricle wall motion abnormality, stress cardiomyopathy, broken heart syndrome, apical ballooning, takotsubo cardiomyopathy

## Abstract

Takotsubo cardiomyopathy (TC) is a rare, reversible cause of left ventricular wall motion abnormality (LVWMA) that mimics the presentation of acute myocardial infarction (AMI). TC is usually preceded by an emotional or physical stressor and appears to be more common in postmenopausal women. Various pathophysiological hypotheses of TC have been proposed, but the exact mechanism of action remains elusive. Elevated levels of catecholamines leading to cardiac dysfunction are the most prevalent hypothesis. The protective role of estrogen in the development of cardiomyopathies has been studied extensively. International Takotsubo Diagnostic Criteria (InterTAK) and Mayo clinic diagnostic criteria both have the stipulation stating prevalence of TC is higher in postmenopausal women which hints towards the protective role of estrogen in the development of TC. To review the protective role of estrogen in the mechanism of this novel pathology, we searched Pubmed and Google scholar for the relevant articles by using keywords such as: “takotsubo cardiomyopathy”, “apical ballooning”, “broken heart syndrome”, “stress cardiomyopathy”, “left ventricle wall motion abnormality”, “estrogen”, “estradiol” and “sex hormones”. Our research revealed that although the prevalence of TC is greater in postmenopausal women as compared to men, the prognosis is worse in men. It also revealed the involvement of multiple cellular pathways under the influence of estrogen that could explain the cardioprotective effect of estrogen. Most of the articles found were based on animal studies, thus, there is an emphasis on future human studies. However, we strongly suggest evaluating estrogen levels as part of the initial workup for any patient presenting with signs and symptoms of cardiac pathology.

## Introduction and background

Epidemiology

The prevalence of takotsubo cardiomyopathy (TC) is 1%-2% in patients presenting with symptoms of acute coronary syndrome (ACS) [1]. Brinjikji et al. (2012) studied the in-hospital mortality rate among patients with TC. The study included a sample of 24,701 patients from the National Inpatient Database Samples between 2008-2009 and revealed a mortality rate of 4.2% in patients presenting with TC. Stratifying the study by gender revealed that 89% of patients with TC were females, with a higher mortality rate in men as compared to women (8.4% vs 3.6%) [2]. Another cohort study by Stiermaier et al. (2016) studied the long-term mortality rate of patients with TC in comparison to the mortality rate of patients with ST-elevation myocardial infarction (STEMI). After studying a total of 286 patients, the long-term mortality rate was found to be worse in patients with TC as compared to patients with STEMI (24.7% versus 15.1%, hazard ratio (HR) 1.58, 95% confidence interval (CI) 1.07-2.33) [3]. Because of the apparent cardioprotective role of estrogen on cardiomyopathies, it is of great importance to have a thorough understanding of the underlying pathophysiology of this novel disease along with the cellular changes that occur under the influence of estrogen.

Background

**Figure 1:**
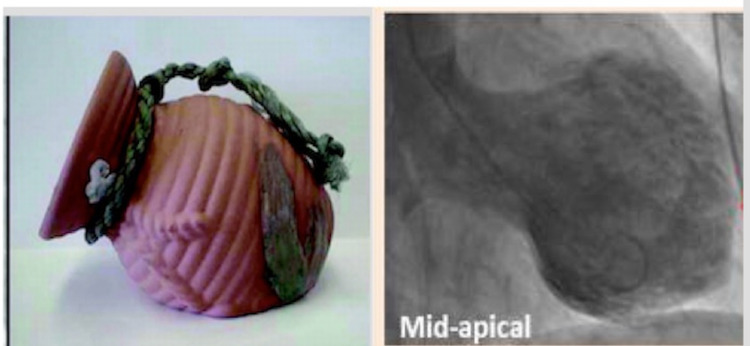
Pictorial representation of octopus catcher vs apical ballooning of left ventricle Modified picture representing the shape of octopus catcher (left) compared to the apical ballooning of left ventricle under stress (right). Y-Hassan S, Tornvall P et al. [5]

TC is a rare disease that has recently started to gain recognition. TC was first described by Satoh (1990) in Japan who derived the name of the pathology from the shape of the container which is used to catch octopus (tako=octopus, tsubo=pot) [4]. The shape of the container is rounded at the bottom with a thin neck, representing the shape of the left ventricle in TC [4-5]. Another name of the disease is “apical ballooning”, which represents the shape of the left ventricle during systole. Although apical ballooning of the left ventricle is the most common finding in TC, other left ventricle wall motion abnormalities (LVWMA) can also be found at the midapical, midventricular, midbasal, and basal regions. The presentation of TC is similar to someone experiencing acute myocardial infarction (AMI), such that the patient presents with chest pain, palpitations, hypertension, light-headedness, and elevated troponin I. However, unlike AMI, patients with TC do not have coronary artery disease (CAD) and the disease is reversible [4-5]. Figure 1 below shows the shape of the octopus catcher and the shape of the left ventricle under stress, thus, the name takotsubo-cardiomyopathy.

The exact pathophysiology of TC is unknown. The most widely accepted hypothesis is the elevated level of catecholamines is preceded by an emotional or physical trigger, as presented by Wittstein et al. (2005) [6]. The study evaluated 19 patients with left ventricle dysfunction after emotional stress, and 13 patients showed elevated levels of catecholamines. The study also observed complete resolution of left ventricular dysfunction. Furthermore, the histopathological analysis of TC myocardium showed contraction band necrosis, in contrast to coagulation necrosis seen in myocardial infarction (MI) [6].

Numerous diagnostic criteria have been proposed due to the novelty and complexity of the disease. In 2008, Mayo Clinic proposed a revised diagnostic criteria that had been widely used [7]. Ghadri et al. (2018) proposed the International Takotsubo Diagnostic Criteria (InterTAK criteria) [7]. According to the InterTAK criteria, patients display transient apical left ventricle ballooning, an emotional or physical stressor acting as a trigger, neurologic disorder including pheochromocytoma acting as a trigger, new ECG changes (ST-segment elevation, ST-segment depression, T- wave inversion, QTc prolongation), moderate elevation of cardiac biomarkers, having a CAD which is not a contraindication to TC, no evidence of infectious myocarditis, and being a postmenopausal woman [7].

The predominance of TC in postmenopausal women suggests the protective role of estrogen. It has been well established by Ostadal et al. (2014) that the incidence of ischemic heart disease (IHD) is lower in premenopausal women as compared to postmenopausal women [8]. Ostada et al. (1984) conducted an observational study to gain insight into the impact of estrogen on the heart with oxygen deprivation. The study demonstrated that the male rat heart was less tolerable to oxygen deprivation than the female rat heart [9].

This review article aims to further explore the cardioprotective effect of estrogen. We aimed to reveal the changes that occur at the cellular level under the influence of estrogen and the estrogen-mediated effects through three kinds of estrogen receptors such as estrogen receptor- alpha (ER-ɑ), estrogen receptor- beta (ER-β) and G-protein coupled estrogen receptor (GPER). In this review article, we searched Pubmed and Google scholar using the following keywords: “takotsubo cardiomyopathy”, “apical ballooning”, “stress cardiomyopathy”, “ broken-heart syndrome”, “cardiomyopathy”, “ estrogen”, “sex hormones” and “ estradiol”. Our search revealed 181 articles. After screening the title and abstract of each article from the year 2000 onwards, we included 20 articles that directly discussed the effects of estrogen and its receptors. In this review, we also discuss the impact of each type of ER individually and mention their protective effect against TC.

## Review

Estrogen is a sex hormone produced by the ovaries and is responsible for the development of secondary sexual characteristics in females. It has been well established that premenopausal women have a lower incidence of cardiac events as compared to men [10]. Also, the rate of IHD increases in postmenopausal women as compared to pre-menopausal women [8]. Estrogen exerts its physiological response by binding to its receptor, ER. Traditionally, two types of ER, ER-ɑ and ER-β, were thought to mediate the response of estrogen. However, the third type of receptor, GPER, has also been found recently to play a significant role in the cardioprotective mechanism of estrogen.

A breakthrough study to mimic the presentation of TC in vivo was done by Ueyama et al. (2007) [11]. Their mouse model was ovariectomized (OVX) and treated with estrogen (OVX+E) to study the cellular response to immobilization (IMO) stress. After the induction of IMO, the heart rate was higher in OVX as compared to OVX+E. Furthermore, the study elucidated the involvement of sympathoadrenal pathways and found elevated levels of c-fos mRNA, a marker for cellular activation in OVX as compared to OVX+E. Also, they were successfully able to demonstrate the expression of both ER-ɑ and ER-β receptors in estrogen-treated models which led to the increased expression of cardioprotective heat shock protein 70 (HSP 70) and atrial natriuretic peptide (ANP). Therefore, this study strongly suggests the cardioprotective role of estrogen and may help explain the increased incidence of TC in postmenopausal women as compared to pre-menopausal women [11].

To understand the association of estrogen and ischemic/reperfusion (I/R) injury, Booth et al. (2005) designed an experimental study where they studied the cardioprotective role of ER-ɑ and ER-β on female rabbit hearts [12]. After pretreating the specimen with ER-agonists (PPT:ER-ɑ and DPT:ER-β) for 30 minutes, they induced a 30-minute ischemic injury by occluding the left anterior descending artery followed by four hours of reperfusion and then measured the infarct size. They observed that the infarct area of ER-ɑ receptors was of a smaller size as compared to ER-β receptors, indicating that the ER-ɑ receptor might play a major role in the cardioprotective effect [12]. In another study, Pelzer et al. (2005) studied ER-β knockout in OVX mice [13]. They induced MI and found increased mortality in knockout mice as compared to wild type (WT) [13]. We can infer from these studies that ER-ɑ is predominantly cardioprotective for AMI, whereas ER-β might play a role in lowering mortality.

The mitochondria play a vital role in the overall integrity of a cell. Mitochondrial connexin43 (Cx43) is crucial to cardiac myocyte recovery following I/R injury [14]. Wang et al. (2019) studied the response of estrogen (17β estradiol, E2) on the Cx43 using cardiomyocytes from a mouse model. Using immunoelectron microscopy and co-immunoprecipitation to determine the interaction of mitochondrial Cx43 with E2, myocardial function, and infarct size were measured. E2 with Cx43 improved the functional recovery of mitochondria and also improved the infarct size in female rats as compared to untreated rats [14].

To elaborate on the association of estrogen and congestive heart failure (CHF), Satoh et al. (2007) studied the estrogen-induced protection against CHF [15]. After eight weeks of estrogen treatment, they found the transgenic mice had improved contractility with decreased cardiac hypertrophy. Decreased apoptosis was observed with reduced Rac1 pathway activity along with increased thioredoxin and decreased thioredoxin reductase activity correlating with increased antioxidative properties of E2, thereby, providing further cardioprotection [15]. 

Zhang et al. (2018) studied the therapeutic properties of EXD (Er-Xian Decoction), commonly found in Chinese medicine to treat menopausal syndrome [16]. After 12 weeks of treatment with EXD, a substantial improvement in cardiac function was observed. The Kyoto Encyclopedia of Genes and Genomes (KEGG) analysis showed expression of genes in the ventricle which could play a part in decreasing the expression of mRNA, myosin, and integrin, thereby helping with cardiac contractility [16].

The counterplay of estrogen and progesterone in relation to myocardial I/R insult was studied by Booth et al. (2007) [17]. Infarct size was measured after the hypoxic insult and revealed a significant small infarct size in estrogen-treated models as compared to OVX mice. In addition, the reversal of the observed cardioprotection was seen under the influence of progesterone [17]. 

Other studies done by Xu et al. (2004) and Luo et al. (2016) found estrogen-treated models to exhibit decreased expression of nicotinamide adenine dinucleotide phosphate (NADPH) oxidase and decreased production of reactive oxygen species (ROS) respectively [18,19], which further shines a light on the cardioprotective impact of estrogen. Table 1 summarizes the findings of all the above-mentioned studies.

**Table 1:** Cardioprotective role of estrogen receptors E2: estrogen or 17B-estradiol; EXD: Er-Xian Decoction; Trx: thioredoxin; MnSOD: manganese superoxide dismutase; OVX: ovariectomized rats; GPER: G-protein coupled estrogen receptor; ROS: reactive oxygen species; CHF: congestive heart failure; mRNA: messenger ribonucleic acid; NADPH: nicotinamide adenine dinucleotide phosphate; p38β: mitogen-activated protein kinase; c-fos mRNA: cell activation marker; HSP70: heat shock protein; ANP: atrial natriuretic peptide; Cx43: mitochondrial connexin43.

Author	Year	Estrogen/Estrogen receptor	Model	Pathway/Markers studied	Findings	Summary
Uyema et al. [11]	2007	ER-ɑ and ER-β	Ovariectomized mice	c-fos mRNA	E2→ ↑c-fos mRNA, ↑HSP70 and ↑ANP	Estrogen provides cardioprotection via heat shock proteins
Booth et al. [12]	2005	E2, ER-ɑ and ER-β	Rabbit hearts	Infarct size	E2→ ↓infarct size	Estrogen is cardioprotective in ischemic injury
Pelzer et al. [13]	2005	ER-β	ER-β knockout	mortality	ER-β knockout → ↑mortality	Estrogen could be beneficial in lowering mortality
Wang et al. [14]	2019	17β estradiol, E2		Cx43	E2→ ↑Cx43→ ↑Mitochondrial stability → ↓infarct size	Estrogen plays a vital role in stability of mitochondrial integrity
Satoh et al. [15]	2007	17-B Estradiol	G ɑ q transgenic mice	Rac1/ Trx, Trx reductase	↑Trx, ↓Rac1	Treatment with E2 improves CHF
Zhang et al. [16]	2018	EXD	OVX	Myosin, integrin, mRNA	↓mRNA, ↓myosin, ↓integrin	EXD is cardioprotective
Booth et al. [17]	2007	Estrogen & Progesterone	New Zealand mice	Infarct size	↓ Troponin I in Estrogen alone	Cardioprotective effect of E2 reversed by medroxyprogesterone
Xu et al. [18]	2004	E2	Sprague-Dawley rat	Estrogen + Superoxide dismutase mimetic	↓ expression of NADPH oxidase	Superoxide inhibition improves cardiac function
Luo et al. [19]	2016	E2	Neonatal rat cardiomyocyte	P38β, MnSOD	↓ROS	E2 → P38β and MnSOD activation→ ↓ROS

Estrogen receptor-ɑ (E2-ɑ or ER-ɑ)

In an experimental study to understand the cardioprotective effect of E2-ɑ receptors, Xue B et al. (2007) studied the role of E2 in angiotensin II (ANG-II) induced hypertension [20]. After the administration of ANG-II, elevated blood pressures were observed in OVX and in estrogen receptor ɑ knockout (ERɑKO) mice. The mice were then administered estrogen which showed a significant decrease in blood pressure. This cardioprotective effect of estrogen was then reversed after the coadministration of nonselective estrogen antagonists, ICI 182, 780 [20].

Wang et al. (2006) studied the role of ER-ɑ in MI [21]. MI causes the release of proinflammatory cytokines and increases the activation of the mitogen-activated protein kinase (MAPK) family and Jun N-terminal kinase (JNK) pathway which are markers for myocardial dysfunction [21]. Conversely, activation of the extracellular-signal-regulated kinase (ERK) pathway is consistent with cardiac function recovery [21]. Using enzyme-linked immunoassay (ELISA) and western blot techniques, ER-ɑ was found to increase the activation of ERK1/2 and decrease the activation of proapoptotic JNK. Therefore, ER-ɑ may play a major cardioprotective role in MI [21].

Left ventricular outflow tract (LVOT) obstruction occurs in 15%-25% of TC patients leading to heart failure [22]. Westphal et al. (2012) studied the effect of E2, 16-ɑ LE2 (selective E2-ɑ agonist), and raloxifene on transverse aortic constriction (TAC) induced myocardial hypertrophy. After nine weeks, they observed reduced ejection fraction in all the groups, however, E2 and 16-ɑ LE2 administered groups showed slowed progression of systolic dysfunction as compared to the raloxifene administered group, which showed the highest level of myocardial hypertrophy [23]. Also, E2 and 16-ɑ LE2 groups showed decreased fibrosis [23]. Therefore, these studies shine a light on the cardioprotective role of ER-ɑ which might be helpful in developing therapeutic drugs specific for alpha receptors. Table 2 summarizes the findings of the above-mentioned studies. 

**Table 2:** Cardioprotective role of estrogen receptor-ɑ (E2-ɑ) MAPK: mitogen-activated protein kinase; ERK: extracellular-signal-regulated kinase; MH: myocardial hypertrophy; ANG-II: angiotensin II; BP: blood pressure; OVX WT: ovariectomized wild type; ERɑKO: estrogen receptor-ɑ knockout; E2ɑ: estrogen receptor-alpha; 16-ɑ LE2: specific estrogen receptor-ɑ agonist.

Author	Year	Receptor	Pathway/Marker	Cardiovascular pathology	Findings	Conclusion
Xue B et al. [20]	2007	E2-ɑ	Blood pressure	Effect on blood pressure	ANG-II→ ↑ BP in OVX WT and ERɑKO E2→↓BP in OVX WT and ERɑKO	E2ɑ is cardioprotective against hypertension
Wang et al. [21]	2006	E2- ɑ	MAPK and ERK	Myocardial ischemia	E2ɑKO → ↑MAPK, ↓ERK→ myocardial injury	E2ɑ is protective in myocardial ischemia
Westphal et al. [23]	2012	E2-ɑ		MH	E2 and 16-ɑ LE2 → slowed progression of MH	Estrogen helps with myocardial hypertrophy

Estrogen receptor-β (ER-β or E2-β)

Multiple studies have been done to understand the cardioprotective role of ER-β. In one of the studies, Zhu et al. (2002) studied the effect of E2-β on hypertension [24]. In their study model, the vascular smooth muscles of ER-β deficient mice showed increased vasoconstriction and decreased production of inducible nitric oxide synthase (iNOS) as compared to the WT. Conversely, when treated with estrogen, E2-β deficient mice showed reduced vasoconstriction [24].

To further elucidate the role of E2-β on lowering blood pressure, Jazbutyte et al. (2008) treated the OVX mice with E2-β agonist (8β-VE2) [25]. After 12 weeks of treatment, they found an enhanced expression of E2-β in the aorta with improved nitric oxide (NO)-induced vasodilation [25].

Moreover, I/R injury leads to mitochondrial instability by the activation of pro-apoptotic cytochrome c, which in turn causes the activation of caspase 9 [26]. Schubert et al. (2016) demonstrated the protective role of E2-β against mitochondrial instability [27]. They pre-treated the OVX mice with either E2 or ER-β specific agonist (ERβA) and found low levels of cytochrome c and high levels of anti-apoptotic Bcl2 protein [27].

Another study done by Skavdahl et al. (2005) studied the role of E2-β in cardiac hypertrophy [28]. Upon inducing stress via TAC, they observed that mice with genotype of -/-E2-ɑ showed the same response to stress as WT females mice. Also, females with genotype of -/-E2-β showed a significant increase in cardiac hypertrophy, thus, providing concrete data that ER-β plays a significant role in attenuating cardiac hypertrophy [28]. In the same study, Skavdahl et al. (2005) also elaborated on the importance of E2-β on the development of heart failure with reduced ejection fraction (HFrEF) [28]. By measuring the ratio of heart weight to body weight (HW/BW), they found that ERɑKO male mice had the same HW/BW ratio compared to their WT counterparts. Whereas ERβKO mice showed a significant increase in HW/BW ratio [28].

Cardiac hypertrophy is a precursor to diastolic dysfunction, and E2 has been associated with preventing cardiac hypertrophy [29]. Pedram et al. (2008) demonstrated the protective effect of E2, in particular E2-β, on cardiac hypertrophy [29]. After infusing the animal models with ANG-II, which is a known potent vasoconstrictor, they found that animals lacking E2-β showed increased hypertrophy as compared to WT and E2-ɑ KO, thus, indicating the important role of E2-β on cardiac hypertrophy, whereas E2-ɑ did not seem to play a significant role in cardiac hypertrophy [29]. Table 3 summarizes the studies and their findings specific to ER-β.

**Table 3:** Cardioprotective role of estrogen receptor-β (ER-β) iNOS: inducible nitric oxide synthase; HTN: hypertension; I/R: ischemia/reperfusion injury; HW/BW: heart weight/body weight; HFrEF: heart failure with reduced ejection fraction; Bcl 2: B-cell lymphoma 2; ANG-II: angiotensin-II; WT: wild type

Author	Year	Receptor	Pathway/Marker	Cardiovascular pathology	Findings	Summary
Zhu et al. [24]	2002	E2-β	iNOS	HTN	E2-β deficient→↑vasoconstriction, ↓ iNPS production	Lack of E2- β plays role in vasoconstriction
Jazbutyte et al. [25]	2008	E2-β	8β-VE2	HTN	E2-β agonists→ vasodilation	Administration of E2-β agonist improves vasodilation
Schubert et al. [27]	2016	E2-β	Cytochrome c, Bcl 2	I/R injury	E2/ERβA → ↑Bcl 2 and ↓cytochrome c, ↓caspase 9	E2-β improves mitochondrial apoptosis
Skavdahl et al. [28]	2005	E2-β	Hypertrophy	Cardiac hypertrophy HFrEF	-/-E2-β→ ↑hypertrophy -/-E2-ɑ→ same response as WT females -/-E2-ɑ→ same HW/BW as +/+E2 ɑ -/-E2-β→ ↑ HW/BW	E2-β plays a significant role in cardiac hypertrophy E2-β has beneficial role in heart failure with reduced ejection fraction
Pedram et al. [29]	2008	E2-β		Cardiac hypertrophy	ANG-II → ↑ hypertrophy in ERβKO ANG-II→ decreased hypertrophy in WT and E2ɑKO	E2-β but not E2-ɑ is cardioprotective against hypertrophy

GPER

Traditionally, it was thought that estrogen exerts its cellular response only through nuclear receptors, E-ɑ and E-β. However, recently a third kind of ER, GPER, has been found located in the endoplasmic reticulum that binds specifically to estrogen to produce nongenomic effects of estrogen [30]. Fredette et al. (2017) studied the involvement of GPER in the production of vasodilatory NO in human endothelial cells. After treating endothelial cells with non-selective ER, the study found a five-fold increase in the production of NO relative to basal NO [31]. Furthermore, treatment with G-1, a selective GPER agonist constituted 73% of NO production by E2 [31]. In addition, treatment with a G-1 selective antagonist reduced the NO production by 58%. The study also found that the activation of GPER led to the production of endothelial nitric oxide synthase (eNOS) which in turn activated a cascade of signaling pathways. eNOS activation by GPER involves PI3/Akt and ERK1/2 pathways, whereas the vasodilatory response involves s-Src/EGFR pathways [31]. Since estrogen is found to be protective against atherosclerosis, Meyer et al. (2014) studied the role of GPER in the progression of atherosclerosis [32]. In their G-protein coupled estrogen receptor knock-out (GPERKO) mouse model, they found that there was decreased production of vasodilatory NO and increased production of LDL, which led to the progression of atherosclerosis [32]. In another study, Deschamps et al. (2009) studied the cardioprotective role of GPER in I/R injury [33]. They found that the acute activation of GPER with G-1 was directly responsible for the activation of the PI3K/Akt pathway, which has cardioprotective properties [33]. To further delve into the mechanism of cardioprotection against I/R by GPER, Bopassa et al. (2010) designed an experimental study where they studied the G-1 induced cellular response in isolated mice hearts using the Langendorff technique [34]. Under stress, there was an increased calcium influx which caused an increased mitochondrial permeability transition pore (mPTP) opening. When treated with G-1, there was a decreased infarct size, improved cardiac function, and above all, decreased mPTP [34]. They also observed an increase in phosphorylation of Erk which led to the conclusion that the cardioprotective role of GPER is mediated through the Erk pathway. To further illustrate the role of Erk, they treated cardiac myocytes with PD (Erk inhibitor) which abolished the cardioprotective effect of G-1 [34]. Kang et al. (2012) studied the role of GPER in heart failure [35]. They induced heart failure in Sprague-Dawley rats with isoproterenol (ISO) and found a decrease in cardiac function, increase in fibrosis, increase in brain natriuretic peptide (BNP), and decrease in phosphorylated Akt in the OVX+ISO group, which was reversed in G-1 treated mice [35].

Table 4 provides the summary of the above-mentioned studies elucidating the specific cardioprotective role of GPER.

**Table 4:** Cardioprotective role of GPER GPER: G-protein coupled estrogen receptor: eNOS: endothelial nitric oxide synthase; G-1: selective GPER agonist; GPERKO: G-protein coupled receptor knockout; Akt: protein kinase B; PI3K: phosphoinositide-3-kinase; LDL: low-density lipoproteins; mPTP: mitochondrial permeability transition pore; PD: Erk inhibitor; HTN: hypertension; NO: nitric oxide; ISO: immobilization stress; ERK: extracellular-signal-regulated kinase.

Author	Year	Receptor	Pathway/Marker	Cardiac pathology	Findings	Summary/Conclusions
Fredette et al. [31]	2017	GPER	eNOS	HTN	G-1→ ↑ eNOS→ ↑ c-Src,ERK1/2, GPER, PI3K/Akt→ ↑ NO production→ ↑ vasodilation→ ↓ Hypertension	GPER plays a vital role in NO production, thereby, decreasing HTN
Meyer et al. [32]	2014	GPER		Atherosclerosis	GPERKO→ ↓ NO production, ↑ LDL production, ↑ inflammation	GPER plays a major role in progression of atherosclerosis
Deschamps et al. [33]	2009	GPER	Sprague-Dawley rat	Akt, ERK1/2, PI3K	↓ infarct size, ↓ postischemic contractile dysfunction	Akt and ERK 1/2 pathways are protective;PI3K reverses the protection
Bopassa et al. [34]	2010	GPER	mPTP	I/R	G-1 → ↓mPTP, ↓infarct size,↑ Erk→ ↓cell death G-1+PD→ ↑mPTP,↑infarct size, ↓Erk → ↑cell death	GPER protects against ischemia/reperfusion injury
Kang et al. [35]	2012	GPER		Heart failure	ISO→ ↓ejection fraction G-1→ ↑ejection fraction	GPER improves ejection fraction

## Conclusions

TC is a reversible cardiomyopathy that impacts men and postmenopausal women more than premenopausal women. Estrogen seems to have a cardioprotective role in cardiomyopathies with a complex mechanism of action. The purpose of this article was to have a thorough understanding of this novel pathology. The studies mentioned in this article present us with strong pieces of evidence towards the cardioprotective role of estrogen with a positive impact on infarct size, cardiac hypertrophy, hypertension, heart failure, etc. A major limitation of this article is that all the studies included in this review are animal based, thus, there is a need to develop human studies to extrapolate these findings. Although estrogen replacement therapy has been known to be beneficial against vaginal atrophy and osteoporosis, a significant side effect of estrogen supplement treatment is the development of a hypercoagulable state resulting in deep vein thrombosis and potentially pulmonary embolism. Therefore, we recommend future studies focus on an estrogen regimen that would be beneficial to both sexes. The most prevalent theory about the mechanism of TC is that elevated levels of catecholamines lead to a hyperadrenergic state. However, our research could not find an article linking estrogen and the hyperadrenergic state of catecholamines. In addition, we strongly recommend including estrogen levels as part of the initial blood work for patients presenting with signs and symptoms of cardiac pathology.
